# Awareness of Artificial Intelligence in Medical Imaging Among Radiologists and Radiologic Technologists

**DOI:** 10.7759/cureus.38325

**Published:** 2023-04-30

**Authors:** Kamal Alsultan

**Affiliations:** 1 Department of Diagnostic Radiology Technology, College of Applied Medical Sciences, Taibah University, Medina, SAU

**Keywords:** radiologic technologists, radiologists, medical imaging, artificial intelligence, awareness

## Abstract

Background: Current technological developments in medical imaging are primarily focused on increasing the integration of artificial intelligence (AI) into all medical imaging modalities. They are already considered capable of handling tasks such as image reconstruction, processing (denoising, segmentation), analysis, and predictive modeling. The purpose of this study is to assess the awareness (knowledge, attitudes, and practices) of radiologists and radiologic technologists regarding AI in medical imaging.

Materials and methods: This cross-sectional, qualitative study focuses on radiologists and radiologic technologists in Saudi Arabia, Sudan, and Yemen. A self-administered questionnaire based on published studies was used to collect primary data. Version 25.0 of IBM SPSS Statistics (IBM Corp., Armonk, NY) was used for the statistical analysis. The demographics were summarized as frequency and percentage. Independent samples t-tests and ANOVA tests were used to evaluate and compare the degree of AI awareness among the study groups.

Results: A total of 210 individuals completed the survey. According to demographic information, there were 134 (63.8%) radiologic technologists and 76 radiologists (36.2%). Of the participants, 131 (62%) were male, while 79 (37.6%) were female. A total of 130 (61.9%) of the targeted respondents had a positive attitude, 105 (50%) had appropriate practice, and 122 (58.1%) of them were informed (knowledgeable) about AI in medical imaging. There was a significant difference in knowledge awareness between radiologists and radiologic technologists (p-value: <0.05). Radiologists were more knowledgeable than radiologic technologists, and females were more knowledgeable than males (p-value: 0.049). For attitude awareness, there were no significant differences regarding specialization, gender, age, academic qualification, and experience (p-value > 0.05). Regarding practice awareness, it turned out that females are more knowledgeable than males (p-value: 0.007). Additionally, it was discovered that significant differences indicated that bachelor's degree holders have a higher level of practice awareness than diploma holders (p-value: <0.05).

Conclusion: Significant differences between the respondent's knowledge awareness regarding specialization, gender, and experience are linked with relatively sufficient AI-basic knowledge and positive attitude awareness among radiologists and radiologic technologists. Only half of the study participants had appropriate practical awareness; therefore, additional training could enhance practical awareness.

## Introduction

Artificial intelligence (AI) has the potential to revolutionize healthcare by achieving provider-level performance across a range of medical specializations. Diagnostic medical imaging (DMI) is one of the medical subspecialties that produces the most digital data overall. Every DMI department routinely produces a significant and diverse amount of data. To extract information for screening, diagnosis, treatment planning, and prognosis, daily images acquired with various modalities, such as radiography/fluoroscopy, ultrasound, CT, MRI, angiography, or nuclear medicine, are merged with patients' clinical data [1]. In DMI, the goal of creating such applications is to clinically validate the reports and make them workable for diagnostic practice today since there is less time for diagnosis [2,3]. AI is a revolutionary technology that analyzes complex data using computerized algorithms [4]. Each viewpoint offers helpful information regarding moral concerns that should be considered while designing and creating AI technologies. Additionally, it has been found that radiologists and radiologic technologists need to be made aware of and worried about higher-level concerns like fairness, bias, and health inequities. They are more focused on the role of giant corporations in the healthcare industry [3].

The rapid increase in AI use in computer applications has raised a similar concern about its ability to provide users with understandable or explainable output. Although AI offers several benefits, like with every fantastic advancement, there is also a certain degree of risk [5]. Through investigation and literature review, the development of the human-made brainpower framework globally has many positive results in store. It is anticipated that many applications and programs in medical imaging will continue to develop into more accurate, affordable, and accessible versions going from the bench to the bedside at the current rate of AI facilities [6]. Indeed, the AI revolution will have the most effects on breast and oncologic imaging, as well as thoracic, neuroradiology, and nuclear medicine. Since the start of AI in healthcare, this revolution makes sense because integrated screening programs have provided critical digital data, and machine learning techniques are top candidates for breast imaging. Market forecasts from 2017 indicated that between 2022 and 2027, there would be a boom in innovative AI applications for medical imaging [7].

Previous research has revealed that while radiology residents and radiologists with low AI-specific knowledge are more likely to be afraid, those with intermediate to advanced AI-specific expertise are more likely to have a positive attitude toward AI. Therefore, additional instruction might enhance clinical adoption [8]. A total of 475 valid replies were received; participants showed a favorable attitude toward AI regarding bettering clinical quality, making accurate diagnoses, reducing radiation exposure, and advancing research. However, they voiced worries about using this technology, including fears about job security and the loss of fundamental professional radiographer jobs and abilities [9,10]. At the same time, 1032 radiologists participated in a poll; most radiologists think AI will improve their work within five to 10 years, notably in terms of reduced diagnostic error rates and improved workflow, but they also worry that it would hurt their reputation among other professionals (e.g., referring clinicians). The study believes they can provide insight into radiologists' expectations of AI. It should be considered by all parties (including biomedical and information technology researchers, healthcare administrators, and legislators) to successfully implement AI technology in the radiology workplace [10].

To develop medical imaging domains, academic programs must be advanced, in-service training programs implemented, and AI applied to medical imaging. Studies comparing radiologists' and technologists' awareness of AI are limited in Arab countries due to the lack of literature on AI awareness. The study aims to assess the awareness (knowledge, attitude, and practice) among radiologists and radiologic technologists about AI in medical imaging. The researcher hypothesized that respondents' knowledge, attitudes, and practices about AI in medical imaging differed based on their independent variables, like gender and specialization.

## Materials and methods

Study design and participants

This qualitative cross-sectional study focused on radiologists and radiologic technologists who live in Saudi Arabia, Sudan, and Yemen. Self-administered questionnaires adapted from published studies were utilized to collect primary data [2,5]. SurveyMonkey (Momentive, San Mateo, CA) collected data from 210 participants via an online questionnaire. The researcher surveyed over three weeks (1 December to 21 December 2022). A non-serialized internet link was used to distribute the survey to professional agencies and societies through WhatsApp, Twitter, and Facebook. Weekly reminders were sent out. Academic staff who did not work in a health facility were excluded.

Independent variables

The sociodemographic variables used were: (1) specialization (radiologist vs. radiologic technologist/radiographer); (2) gender (female vs. male); (3) age (20-25 years vs. 26-35 years vs. more than 36 years); (4) academic qualification (diploma vs. Bachelor of Science (B.Sc.) vs. Master of Science (M.Sc.) vs. Doctor of Philosophy (Ph.D.)/Doctor of Medicine (MD)); (5) experience (1-5 years vs. 6-10 years vs. 11-15 years vs. more than 15 years).

Dependent variables

In this survey, participants were asked to answer questions assessing their knowledge, attitude, and practice regarding AI in medical imaging. A five-point Likert scale was used for all responses.

Questionnaire design

A closed-ended questionnaire was designed and validated by qualified specialists (two radiologists, two radiologic technology consultants, two computer science and AI specialists, and one community medicine consultant). After that, a pilot was conducted on 17 radiologists and radiologic technologists (Cronbach's alpha = 0.808). The questionnaire consisted of 20 questions, divided into four sections. The first section contained five questions about demographic data, and the other sections contained 15 questions with multiple-choice answers, using a five-point Likert scale (strongly agree, agree, neutral, disagree, strongly disagree), where appropriate. The second section contained six questions that assessed the fundamental knowledge about AI and machine learning: Q1: "I heard about artificial intelligence and machine learning"; Q2: "Artificial intelligence is the simulation of human intelligence in machines that are programmed to think like humans and mimic their actions"; Q3: "Machine learning is a branch of artificial intelligence that focuses on using data and algorithms to imitate how humans learn"; Q4: "Artificial intelligence can provide optimized image analysis"; Q5: "Artificial intelligence can help in diagnosing diseases efficiently and accurately"; Q6: "Artificial intelligence can reduce medical errors." The third section contained four questions and estimated the respondents’ attitudes toward artificial intelligence in medical imaging: Q1: "With artificial intelligence, humans will do less work"; Q2: "I believe that artificial intelligence has contributed to the development of medical imaging fields"; Q3: "I expect that many radiologists and radiology technologists may lose their work in the future"; Q4: "I believe that in the next 10 years, artificial intelligence will improve and change the field of medical imaging." The fourth section contained only five questions that evaluated the respondents’ practice of AI in medical imaging: Q1: "I worked in conventional radiography departments before using the computer in medical imaging"; Q2: "I have attended some workshops and training courses on the application of artificial intelligence in medical imaging"; Q3: "I use artificial intelligence software for dealing with medical imaging daily"; Q4: "I can help in developing artificial intelligence software/applications to do the required tasks"; Q5: "I can deal with large medical imaging data analytics." Results were downloaded into a CSV file at the end of the survey period.

Ethical consideration

The survey objectives stated that the data collected would be used for research and journal publication. After clarifying the questionnaire's objectives, all respondents agreed to participate. Ethical approval was obtained from the Scientific Research Ethics Committee of the College of Applied Medical Sciences, Taibah University, Saudi Arabia (approval number: 2023/150/303 DRD).

Statistical analysis

Statistical analysis was done using IBM SPSS Statistics version 25.0 (IBM Corp., Armonk, NY). Responses from the last three sections by participant demographics were reported as count (frequency and percentage). Group comparison was tested by independent samples t-test for variables with two groups (specialization and gender), and ANOVA (analysis of variance) test for variables with three or more groups (age, academic qualification, study country, residency country, and experience).

For a more straightforward analysis and statistics, the five-point Likert scale (strongly agree, agree, disagree, neutral, and strongly disagree) was recoded according to the median values into a two-point scale. Knowledge awareness, which has six questions summated to 30, was recoded into another variable regarding its median (above the median (24) was recoded as "knowledgeable," and equal or below the median was recoded as "unknowledgeable"). At the same time, attitude awareness, which has five questions summated to 25, was recoded into another variable regarding its median (above the median (16) was recoded as "positive attitude," and equal or below the median was recoded as "negative attitude"). Practice awareness, which has four questions summated to 20, was recoded into another variable regarding its median (above the median (18) was recoded as "adequate practice," and equal or below the median was recoded as "inadequate practice"). A p-value of < 0.05 was considered statistically significant.

## Results

Respondent demographics

The survey was sent to 350 radiologists and radiologic technologists from Saudi Arabia, Sudan, and Yemen. Only 210 respondents completed the survey (response rate: 60%). The demographic data showed that 76 (36.2%) participants were radiologists, and 134 (63.8%) were radiologist technologists/radiographers. Of the participants, 131 (62%) were males and 79 (37.6%) were females. At the same time, the age groups focused were 44 (21%) from the age group of 20-25 years, 70 (33.3%) from the age group of 26-35 years, and 96 (45.7%) from the age group above 36 years. Respondents were arranged in descending order according to academic qualifications as follows: B.Sc. holders were 70 (33.3%), Ph.D./MD holders were 57 (27.1%), diploma holders were 44 (21%), and M.Sc. holders were 39 (18.6%). Most respondents studied in Yemen (40%), followed by 27.6% in Sudan. Most of them were Yemen residents (45.7%). And 44.8% of them had experience in medical imaging between one and five years, followed by respondents with experience of more than 15 years (20.5%) (Table 1).

**Table 1:** Sociodemographic characteristics of the study participants B.Sc.: Bachelor of Science; M.Sc.: Master of Science; Ph.D.: Doctor of Philosophy; MD: Doctor of Medicine.

Variables	Frequency	Percent (%)
Specialization		
Radiologist	76	36.2
Radiologic technologist/radiographer	134	63.8
Gender		
Male	131	62.4
Female	79	37.6
Age		
20-25 years	44	21.0
26-35 years	70	33.3
More than 36 years	96	45.7
Academic qualification		
Diploma	44	21.0
B.Sc.	70	33.3
M.Sc.	39	18.6
Ph.D./MD	57	27.1
Study country		
Saud Arabia	46	21.9
Sudan	58	27.6
Yemen	84	40.0
Other	22	10.5
Residency country		
Saud Arabia	77	36.7
Sudan	37	17.6
Yemen	96	45.7
Experience		
1-5 years	94	44.8
6-10 years	38	18.1
11-15 years	35	16.7
More than 15 years	43	20.5

Respondent’s awareness of AI in medical imaging

According to Table 2, 122 (58.1%) of the respondents were informed (knowledgeable) about AI in medical imaging, 130 (61.9%) of the targeted respondents had a positive attitude, and 105 (50%) of them had adequate practice.

**Table 2:** Respondents’ knowledge, attitude, and practice of artificial intelligence in medical imaging

		N (%)	Mean	Std. deviation
Knowledge	Knowledgeable	122 (58.1)	1.410	0.495
	Unknowledgeable	88 (41.9)		
Attitude	Positive attitude	130 (61.9)	1.381	0.487
	Negative attitude	80 (38.1)		
Practice	Adequate practice	105 (50)	1.5	0.501
	Inadequate practice	105 (50)		

Differences in AI awareness (knowledge, attitude, and practice) of medical imaging among respondents’ specializations and gender

An independent sample t-test was conducted to compare the knowledge awareness about AI of radiologists and radiologic technologists; there was a significant difference (t (149.155) = 3.577, p = 0.000) in the scores with the mean score for radiologists (M = 1.5789, SD = 0.49701) higher than radiologic technologists (M = 1.3284, SD = 0.47138). The magnitude of the differences in the means was as follows: mean difference = 0.25059 and 95% CI: 0.11215 to 0.38903. At the same time, the mentioned test was conducted to compare the attitude awareness toward AI for radiologists and radiologic technologists; there was no significant difference (t (149.649) = 1.473, p = 0.143) in the scores with the mean score for radiologists (M = 1.4474, SD = 0.50053) and radiologic technologists (M = 1.3433, SD = 0.47659). The magnitude of the differences in the means was as follows: mean difference = 0.10408 and 95% CI: -0.03552 to 0.24369. At the same time, in comparing the practice of AI for radiologists and radiologic technologists, an independent sample t-test was conducted; there was no significant difference (t (208) = 0.859, p = 0.391) in the scores with the mean score for radiologists (M = 1.5395, SD = 0.50175) and radiologic technologists (M = 1.4776, SD = 0.50137). The magnitude of the differences in the means was as follows: mean difference = 0.06186 and 95% CI: -0.08011 to 0.20384). Also, an independent sample t-test was conducted to compare the knowledge awareness about AI of males and females. There was a significant difference (t (159.446) = -1.981, p = 0.49) in the scores, with the mean score for females (M = 1.5063, SD = 0.50315) higher than males (M = 1.3664, SD = 0.48367). The magnitude of the differences in the means was as follows: mean difference = -0.13992 and 95% CI: -0.27943 to -0.00040. Also, the test was used to compare the attitude awareness about AI of males and females. There was no significant difference (t (208) = -0.264, p = 0.792) in the scores with the mean score for males (M = 1.3740, SD = 0.48573) and females (M = 1.3924, SD = 0.49141). The magnitude of the differences in the means was as follows: mean difference = -0.01836 and 95% CI: -0.15537 to 0.11865. Also, the test was conducted to compare the practice awareness about AI of males and females. There was a significant difference (t (208) = -2.742, p = 0.007) in the scores with the mean score for males (M = 1.4275, SD = 0.49661) and females (M = 1.6203, SD = 0.48842). The magnitude of the differences in the means was as follows: mean difference = -0.19277 and 95% CI: -0.33138 to -0.05417 (Table 3).

**Table 3:** Results of independent samples t-test show the differences in AI awareness (knowledge, attitude, and practice) between specialization groups (radiologists and radiologist technologists/radiographers), and gender groups (males and females)

Variables	Knowledge		Attitude		Practice	
	Mean (SD)	P-value	Mean (SD)	P-value	Mean (SD)	P-value
Specialization						
Radiologist	1.5789 (0.49701)	0.000	1.4474 (0.50053)	0.143	1.5395 (0.50175)	0.391
Radiologic technologist/radiographer	1.3284 (0.47138)		1.3433 (0.47659)		1.4776 (0.50137)	
Gender						
Male	1.3664 (0.48367)	0.049	1.3740 (0.48573)	0.792	1.4275 (0.49661)	0.007
Female	1.5063 (0.50315)		1.3924 (0.49141)		1.6203 (0.48842)	

Differences in AI awareness (knowledge, attitude, and practice) of medical imaging among respondents’ age, academic qualification, and experience

The hypothesis tests if the awareness of AI in medical imaging differs across age and experience. The ANOVA results suggest that the age groups' knowledge, attitude, and practice awareness scores do not vary significantly (knowledge: F2,207 = 0.942, p = 0.391; attitude: F2,207 = 0.942, p = 0.783; practice: F2,207 = 0.942, p = 0.226). At the same time, ANOVA results suggest that the knowledge and attitude awareness scores of the academic qualifications do not differ significantly (knowledge: F3,206 = 0.023, p = 0.995; practice: F3,206 = 1.105, p = 0.348), but the test suggests that the practice awareness scores of the academic qualifications differ significantly (F3,206 = 3.660, p = 0.013). To check the individual differences between groups, post-hoc comparisons were assessed using Bonferroni. The test indicated that the mean scores for diploma holders (M = 1.2955, SD = 0.46152) significantly differed from B.Sc. holders (M = 1.6000, SD = 0.49344). The ANOVA results suggest that the knowledge awareness scores of the experience differ significantly (F3,206 = 3.206, p = 0.024). To check the individual differences between groups, post-hoc comparisons were assessed using Bonferroni; the test indicated that the mean scores for the experience group of 11-15 years (M = 1.5429, SD = 0.50543) were significantly different from the experience group of more than 15 years (M = 1.2326, SD = 0.42746). The ANOVA results suggest that the attitude and practice awareness scores of the experience do not differ significantly (attitude: F3,206 = 0.762, p = 0.516; practice: F3,206 = 1.546, p = 0.204) (Table 4).

**Table 4:** Results of one-way ANOVA show the differences in AI awareness (knowledge, attitude, and practice) between age groups, academic qualifications, and experience B.Sc.: Bachelor of Science; M.Sc.: Master of Science; Ph.D.: Doctor of Philosophy; MD: Doctor of Medicine.

Variables	Knowledge		Attitude		Practice	
	Mean (SD)	P-value	Mean (SD)	P-value	Mean (SD)	P-value
Age						
20-25 years	1.3409 (0.47949)	0.391	1.3636 (0.48661)	0.783	1.6136 (0.49254)	0.226
26-35 years	1.4714 (0.50279)		1.4143 (0.49615)		1.4857 (0.50340)	
More than 36 years	1.4167 (0.49559)		1.3646 (0.48384)		1.4583 (0.50088)	
Academic qualification						
Diploma	1.4091 (0.49735)	0.995	1.2727 (0.45051)	0.348	1.2955 (0.46152)	0.013
B.Sc.	1.4143 (0.49615)		1.4286 (0.49844)		1.6000 (0.49344)	
M.Sc.	1.4359 (0.50236)		1.3590 (0.48597)		1.4872 (0.50637)	
Ph.D./MD	1.4211 (0.49812)		1.4211 (0.49812)		1.5439 (0.50250)	
Experience						
1-5 years	1.4681 (0.50166)	0.024	1.4362 (0.49857)	0.516	1.5745 (0.49707)	0.204
6-10 years	1.3947 (0.49536)		1.3421 (0.48078)		1.5000 (0.50671)	
11-15 years	1.5429 (0.50543)		1.3143 (0.47101)		1.4000 (0.49705)	
More than 15 years	1.2326 (0.42746)		1.3488 (0.48224)		1.4186 (0.49917)	

## Discussion

The participants' knowledge, attitude, and practice of AI in medical imaging were studied in the current study. It was observed that half of the participants were aware of AI.

It has been noted that radiologists and radiologic technologists needed stronger knowledge scores on average. This result is consistent with the findings of Tajaldeen and Alghamdi, who stated a severe absence of AI understanding in radiology departments at hospitals in various Saudi Arabian locations [2]. Huisman et al. consistently noted that radiology residents and radiologists need more expertise in AI specifically [8]. A prior study found that 67.2% of radiologists were eager to participate in an AI research project, and 76.0% of radiologists intended to increase their knowledge of AI [11]. At the same time, Rainey et al.’s study showed that radiographers are perceived to lack knowledge, expertise, and confidence when using AI solutions. Still, they also highlight the need for formalized AI education to prepare the current and upcoming workforce for the clinical integration of AI in healthcare and help them navigate the digital future safely and effectively [12]. There needs to be more consistency with Qurashi et al.’s results, which found that the majority of participants (n = 186, 83%) were generally familiar with the function of machine learning and the idea of AI [13]. Radiologists were substantially more aware of AI than radiologic technologists, and there was a significant gender difference. It was observed that females were more knowledgeable than males. This finding contradicts Huisman et al., who reported that males were more knowledgeable than females [8]. This discrepancy might be due to the less sample size of our study.

The result of the finding of this study showed that radiologists and radiologic technologists are the same based on age, academic level, or experience. Regarding the awareness of IA, there is still a knowledge gap. A proportionate amount of training for radiologists and radiologic technologists should accompany the development of AI technologies and their applications.

The study found that more than half of the participants have a positive attitude. Radiologists and technologists hold comparable attitudes toward AI. This result corroborated a prior study by Waymel et al. on radiologists' perceptions of AI in radiography, which revealed a generally favorable attitude toward AI [14]. Furthermore, Coakley et al. found that most radiographers were enthusiastic and had a positive attitude toward the growth of AI [15]. Therefore, no discrepancy exists between radiologists and technologists.

The study also revealed no significant difference between radiologists and radiologic technologists according to specialization, gender, age, academic qualification, and experience. The target audience's impressions help to improve the integration of AI technologies into medical imaging and benefit from its capabilities in offering highly efficient medical diagnostic services.

The study revealed that half of the target population employs the adequate practice of AI processes. This percentage needs to be more vital regarding the practice awareness of AI. However, a study in Africa reported that the awareness of AI in medical imaging practice had been recognized. Job security and data protection issues must be seriously considered to effectively adopt these cutting-edge technologies in African medical imaging [9]. There was a significant difference in practice awareness based on gender, with females performing better than males, while B.Sc. holders performing better than diploma holders. These results supported that education and gender difference are influencing factors for the practice awareness of AI.

The study's limitation was the small number of participants who responded to the qualitative questionnaire, and it was not anticipated that every participant would answer. Furthermore, telephone interviews were not conducted, which could have helped further participants with more in-depth information on viewpoints due to poor internet connectivity and communication difficulties. Additionally, more studies are needed to compare the responses of radiologists and radiologic technologists at the same time.

## Conclusions

The study concluded that most radiologists and radiologic technologists have a fundamental knowledge and positive attitude about AI in medical imaging. Still, only half of them are aware of its practice awareness. The findings also revealed a significant knowledge gap, with radiologists having more knowledge than radiologic technologists and females being more knowledgeable than males. There were no significant differences between the respondent's specialty, gender, age, academic qualification, and experience, according to attitude awareness. There is an urgent need to raise awareness regarding AI in several diagnostic domains due to its rapid growth and application in diagnostic medical imaging.
